# Tackling a Post-COVID-19 Cholecystectomy Waiting List: Are We Meeting the Challenge?

**DOI:** 10.3390/medicina59101872

**Published:** 2023-10-21

**Authors:** Mohammed Hamid, Neginsadat Mirtorabi, Abdul Ghumman, Ayesha Khalid, Mohamed Saleem Noormohamed, Spyridon Kapoulas, Rishi Singhal, Rajwinder Nijjar, Martin Richardson, Tom Wiggins

**Affiliations:** University Hospitals Birmingham NHS Foundation Trust, Heartlands Hospital, Birmingham B9 5SS, UK

**Keywords:** waiting list (MeSH), cholecystectomy (MeSH), cholecystectomy, laparoscopic (MeSH), COVID-19 pandemic, service recovery

## Abstract

*Background and Objectives*: The COVID-19 pandemic has led to a tremendous backlog in elective surgical activity. Our hospital trust adopted an innovative approach to dealing with elective waiting times for cholecystectomy during the recovery phase from COVID-19. This study aimed to evaluate trends in overall cholecystectomy activity and the effect on waiting times. *Materials and Methods*: A prospective observational study was undertaken, investigating patients who received a cholecystectomy at a large United Kingdom hospital trust between February 2021 and February 2022. There were multiple phased strategies to tackle a 533-patient waiting list: private sector, multiple sites including emergency operating, mobile theatre, and seven-day working. The correlation of determination (R^2^) and Kruskal–Wallis analysis were used to evaluate trends in waiting times across the study period. *Results*: A total of 657 patients underwent a cholecystectomy. The median age was 49 years, 602 (91.6%) patients had an ASA of 1-2, and 494 (75.2%) were female. A total of 30 (4.6%) patients were listed due to gallstone pancreatitis, 380 (57.8%) for symptomatic cholelithiasis, and 228 (34.7%) for calculous cholecystitis. Median waiting times were reduced from 428 days (IQR 373–508) to 49 days (IQR 34–96), R^2^ = 0.654, *p* < 0.001. For pancreatitis specifically, waiting times had decreased from a median of 218 days (IQR 139–239) to 28 (IQR 24–40), R^2^ = 0.613, *p* < 0.001. *Conclusions*: This study demonstrates the methodology utilised to safely and effectively tackle the cholecystectomy waiting list locally. The approach utilised here has potential to be adapted to other units or similar operation types in order to reduce elective waiting times.

## 1. Introduction

The COVID-19 pandemic had a significant impact on health systems around the world, with numerous publications in recent years documenting the increasingly difficult challenges faced by various medical specialties [1]. Healthcare systems required swift restructuring to tackle the influx of patients affected by the virus, directly impacting existing services for chronic and non-urgent conditions, affecting medical training, and necessitating the adaptation of the technology used in providing care [2,3].

The entire public, including health professionals, was confronted daily with images and reports of a worldwide collapse of the healthcare system due to the COVID-19 viral pandemic, especially during the first wave of the pandemic [4]. There was fear of infection and the possibility of transmission through family members and friends, but also fear of a lack of COVID-19 protective clothing for healthcare workers and equipment to treat patients [5]. The overwhelming nature of the pandemic had a significant influence on the psychological and occupational well-being of healthcare workers [6].

Hospital departments minimized or suspended planned admissions and elective surgeries to preserve hospital beds. Despite this, delays in hospital admissions for life-threatening or emergency conditions such as acute myocardial infarction, acute appendicitis and testicular torsion still occurred [7,8,9]. The suspension of elective surgery led to a tremendous worldwide backlog on elective surgical waiting lists.

In England, over seven million people remain on waiting lists for surgery and only 62.2% of patients meet the 18-week elective standard from referral to treatment [10,11,12]. As of October 2022, there were over 400,000 patients who had waited over a year to receive elective surgery [12]. There is an ongoing need for increased surgical capacity to meet demands. NHS England has set a target of providing around 30 percent more elective activity by 2024–25 than before the pandemic [13].

Cholecystectomy is one of the most frequently performed surgical procedures worldwide. Gallstones are responsible for a range of presentations including biliary colic, calculous cholecystitis, obstructive jaundice, and pancreatitis. Data from the early stages of the COVID-19 pandemic indicated there had been a shift towards increased utilisation of conservative management for calculous cholecystitis [14]. This was largely due to the uncertainty surrounding the safety of surgery during these early stages of the COVID-19 pandemic [15]. Due to this suspension of elective surgery, there is now a significant burden of patients awaiting treatment for gallstones. Cholecystectomy has been identified as one of the procedures which should be prioritised based on being a ‘high-volume, low-complexity’ operation which can be performed safely without the need for a large amount of complex infrastructure (such as the requirement for an on-site intensive care unit facility) [16]. Small-scale intensive approaches for the performance of a cholecystectomy have already been proposed [17].

Our hospital trust adopted an innovative approach to dealing with elective cholecystectomy waiting times. This was initiated during the recovery phase, following the initial waves of the COVID-19 pandemic. This study aimed to investigate the methodology of the surgical practice undertaken within our unit to tackle the backlog of cholecystectomies, and to provide an understanding of the influence of these practices upon patient waiting times.

## 2. Methods

### 2.1. Study Design and Setting

A prospective observational study was performed to investigate patients awaiting a cholecystectomy within our trust between 1 February 2021 and 28 February 2022. This study was conducted across multiple sites associated within one of the United Kingdom’s (UK) largest hospital trusts. Local institutional approval was gained before data collection was undertaken (local approval number CARMS-17642, 1 August 2021).

During the pandemic, the Federation of Surgical Specialty Associations provided recommendations on the categorization and prioritization of surgical procedures, which were endorsed by the Royal College of Surgeons of England [18]. Patients with gallbladder disease that were deemed suitable to undergo a cholecystectomy were placed onto the operating waiting list and categorised into groups of urgency based on their presenting pathology and health status (Table 1). The records of the patients on the waiting list from before the pandemic were reviewed by clinical teams and re-categorised according to these criteria based on urgency. Patients diagnosed with gallstone pancreatitis and considered suitable for surgery were categorised under group 2a to meet the recommendation that surgery should be completed within two weeks of presentation [19].

### 2.2. Patient Selection Criteria

An electronic waiting list was generated from the Upper Gastrointestinal Surgery Department for the study period. This included all patients awaiting a cholecystectomy at the start date of the present study. This was updated monthly to capture all patients who had been added to the waiting list. Patients under the age of 16 were excluded.

### 2.3. Data Collection

Data collected included demographics (age, gender, diagnosis, and American Society of Anaesthesiologists (ASA) grade), waiting list information (priority categorisation and waiting time to surgery), operative details (emergency or elective procedure, hospital site, operator grade, duration of surgery, and procedure completed), and outcome data (length of hospital stay (LOS), 30-day readmissions [20], mortality, 30-day post-operative complications categorised by the Clavien-Dindo grade [21]). 

### 2.4. Outcomes

The primary outcome was the effect of our trust-wide strategy upon waiting times for laparoscopic cholecystectomy. A sub-group analysis was also performed to assess the effect upon waiting times for gallstone pancreatitis patients. Secondary outcomes included the performance against key outcome parameters for cholecystectomy, including complication rates, conversion to open surgery, and length of stay [19].

### 2.5. Data Analysis

A run chart was plotted to demonstrate changes to the waiting list over time and display weekly procedural statistics. The Kolmogorov–Smirnov test was utilised to test normality with a *p*-value of less than 0.05. Data were summarised using median and interquartile range (IQR) for continuous variables, and number and percentage for categorical data. The correlation of determination (R^2^) and Kruskal–Wallis analysis were used to evaluate trends in waiting times across the study period. *p*-values of <0.05 were considered statistically significant. GraphPad Prism V9.1.3 (GraphPad Software, LLC., Boston, MA, USA) was used for statistical analysis.

## 3. Results

### 3.1. Overall Data

**Figure 1:**
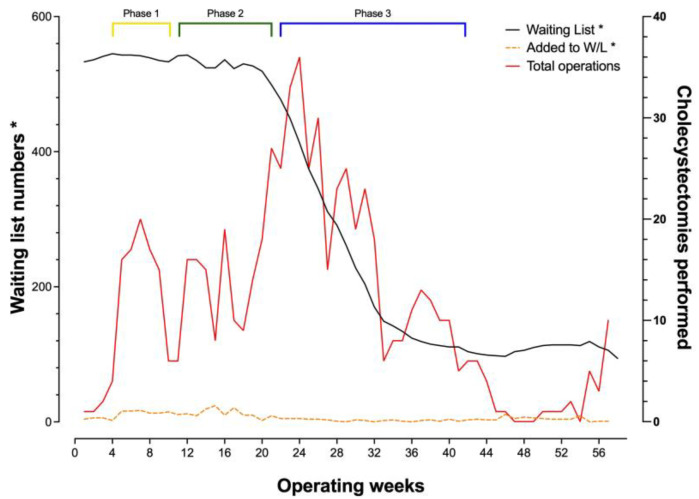
Demonstration of the overall progress with the waiting list (black line) and weekly overall cholecystectomy performance (red line). The keys: “Waiting list*” and “Added to W/L*” correspond to the *y*-axis “Waiting list numbers*”; W/L: Waiting List.

The study period consisted of 56 weeks. There were 533 patients on the waiting list on the first day of the study, and an additional 364 patients added during the study period. There were 145 patients who no longer required surgery for multiple reasons (including patients declining the procedure, other ongoing medical priorities, or failing pre-op assessments due to anaesthetic fitness) and were removed during the course of the study. There was a total of 657 cholecystectomies performed during the study period and by the end of the study the waiting list had reduced to 95 patients (Figure 1).

**Figure 2:**
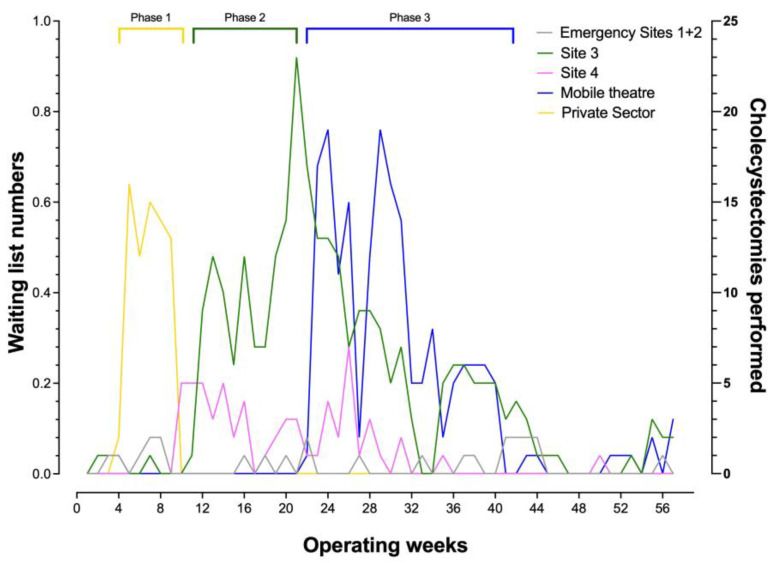
Demonstration of the number of procedures performed in each of the operating theatre locations-during the different phases of the recovery period.

Three distinct operating phases were undertaken during the recovery of the service, with the use of a private sector facility at the outset, alongside two ‘hot sites’ (Sites 1 and 2) where cholecystectomies were performed for patients admitted on an emergency basis (Phase 1, Figure 2). This stage was followed by using an additional trust hospital site (Phase 2, Site 3). Throughout the study period, one other site with a ‘green pathway’ for elective procedures (Site 4) was utilised. Phase 3 involved the utilisation of a mobile operating theatre. Peak performance was observed mid-study during the third phase, with the start of seven-day operating to achieve a maximum of 36 cholecystectomies in one week, outnumbering the new additions to significantly reduce the waiting list numbers. Staffing for the mobile theatre, apart from the anaesthetist and the surgeons, was coordinated by an external contractor linked to the mobile theatre service. Weekend lists were facilitated by internal staff wishing to work additional hours, which prevented disturbances to the normal week-day services. Both services required a robust rota-manager to ensure staffing was supplied for each session well in advance. A disruption to performing cholecystectomies was noted between weeks 44 and 52 when other waiting lists such as hernias and other general surgery procedures were tackled using the same facilities, however this did not significantly impact waiting list numbers for cholecystectomies (black line, Figure 1).

### 3.2. Patient Characteristics

Of the 657 patients who underwent a procedure, 628 (95.6%) were completed electively (Table 2). The median age was 49 years, 602 (91.6%) patients had an ASA of 1-2, and 494 (75.2%) were female. A total of 30 (4.6%) patients were listed for surgery due to gallstone pancreatitis, 380 (57.8%) for cholelithiasis (biliary colic), and 228 (34.7%) for calculous cholecystitis. Trainees were the primary surgeon for 178 (27.1%) of cases. The median length of stay was zero days (IQR 0–1). Just over sixty percent of cases were successfully completed as day cases (399/657—60.7%). The overall median length of stay was zero days (IQR 0-1).

### 3.3. Waiting Times

**Figure 3:**
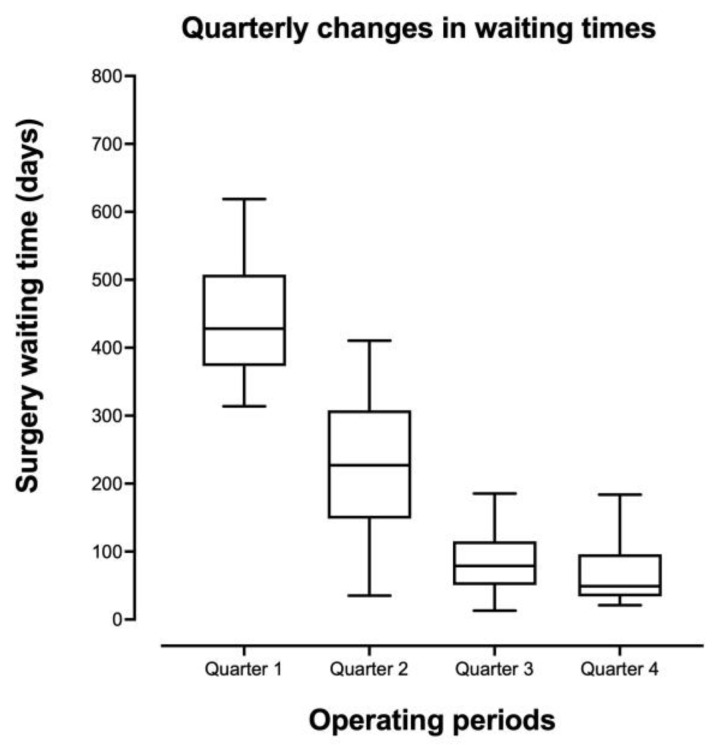
Quarterly changes in elective cholecystectomy waiting times.

**Figure 4:**
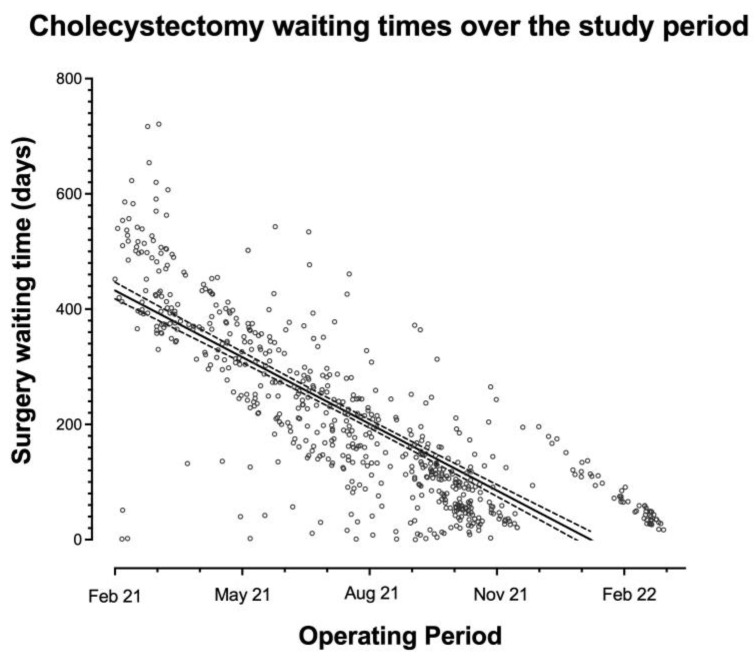
Individual patients’ waiting times over the study period.

**Figure 5:**
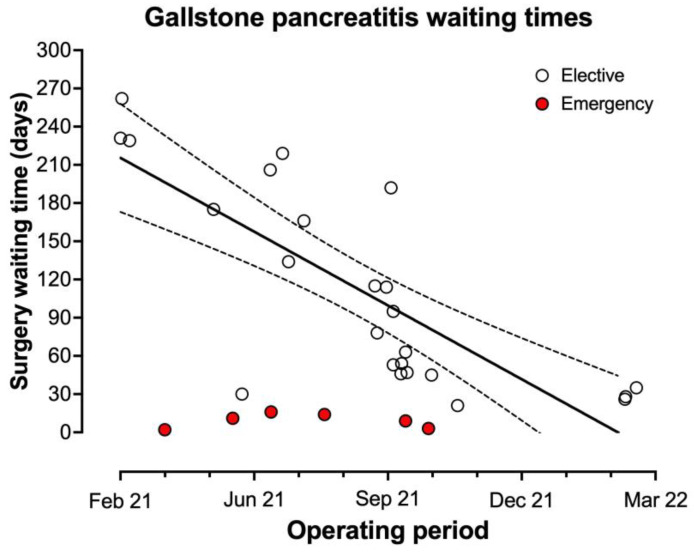
Individual waiting times for gallstone pancreatitis patients.

The median elective waiting times were reduced from 428 days (IQR 373–508) in the first quarter of the study to 49 days (IQR 34–96) in the last quarter, R^2^ = 0.654, *p* < 0.001 (Figure 3 and Figure 4). For pancreatitis specifically, waiting times have dropped from a median of 218 days (IQR 139–239) to 28 days (IQR 24–40) (R^2^ = 0.613, *p* < 0.001 (Figure 5)). 

### 3.4. Readmission and Complications

There were 20 re-admissions within 30 days of surgery (3%). There were 31 total complications (4.7%) with seven being Clavien–Dindo grade three or above (1.1%) [21]. Two patients with retained common bile duct stones required endoscopic retrograde cholangiography (ERCP) (one other retained stone which passed spontaneously was classified as Clavien–Dindo grade two (overall rate of retained bile duct stone 0.5%)). One patient suffered a bile leak requiring a return to theatre. A second patient required a return to theatre due to gallstone ileus following surgery for a complex cholecystogastric fistula (overall re-operation rate 0.3%). One patient was converted to open due to colonic injury identified intra-operatively and there were three other conversions to open surgery (overall rate of conversion 0.6%). There were three intra-abdominal collections which required radiological drainage (0.5%). One patient required unplanned intensive care unit admission due to intra-operative atrial fibrillation with haemodynamic compromise. There were no bile duct injuries and no peri-operative mortalities. Twenty patients had subtotal cholecystectomy, and of these, nine were fenestrated (with four patients having the cystic duct opening closed internally via suture) and eleven non-fenestrated. One of these patients required post-operative ERCP (non-fenestrated group for retained gallstone). Seven cases (1%) were abandoned intraoperatively either due to the extent of intra-abdominal adhesions or significant hepatomegaly.

## 4. Discussion

This study highlights the importance of a phased approach to the recovery of elective surgery and tackling waiting lists. Six primary strategies (priority categorisation, private sector utilisation, multiple surgical sites, emergency operating, mobile theatre environment, and seven-day working) were utilised to effectively reduce the cholecystectomy waiting list. This was successful in decreasing the overall waiting time for surgery from a median of 428 days (IQR 373-508) in the first quarter of the study to just 48 days (IQR 34-96) in the final period of the study. This was achieved whilst also maintaining satisfactory training opportunities, good rates of day-case surgery and low complication rates. 

One key aspect for the development of surgical elective hubs has been to maintain robust governance processes. This ensures care can be delivered to the same standards as expected in other healthcare settings [22]. Within the current dataset, it has been possible to compare results to the key performance indicators (KPI) for laparoscopic cholecystectomy which have been recently published by the British Benign Upper Gastrointestinal Surgical Society (BBUGS) [19]. The present data demonstrate a re-admission rate within 30 days below the KPI target of 10% (3.0% in this series), low conversion rates to open surgery (KPI target below 5%, 0.6% in present series), and acceptable rates of day case surgery (KPI standard 50%, result 60.7% (additional target of 75% not met)). Complication rates were also low with one bile leak (0.2%) (KPI target < 1.5%), three retained stones (0.5%) (KPI target < 2.5%) and no bile duct injuries (KPI target < 0.3%). There were also no peri-operative mortalities in the present series (KPI target <0.1%) [19]. These performance outcomes indicate that this form of surgery can be performed safely in this multi-site model in various surgical environments. Although the KPI target of performing surgery for patients with gallstone pancreatitis within 14 days of the episode [19] has not been achieved here, significant progress was made over the course of the project to reduce the median waiting time for cholecystectomy after gallstone pancreatitis. Prior to the COVID-19 pandemic, our trust had been aiming to meet this target in most cases. However, following the suspension of elective surgery during the COVID-19 pandemic, this wait had dramatically increased with a median wait for surgery for pancreatitis patients of 218 days in February 2021. This was successfully reduced to 28 days by February 2022 through implementing the processes presented in this study. This additional delay beyond 14 days was in part due to issues around the requirement for specific patient testing for COVID-19 during the study period prior to admission to an elective site for surgery. It is anticipated that this waiting time will continue to decrease as surgical capacity continues to develop and the requirements for pre-operative patient screening for COVID-19 are gradually streamlined or lifted [23].

The decrease in elective laparoscopic cholecystectomy during the COVID-19 pandemic has been clearly demonstrated in several large-scale studies from across the globe [24]. However, these same studies have also identified that there was an increase in the utilisation of emergency cholecystectomy during index admission over the same period [24]. Although there is variation in the utilisation of emergency cholecystectomy globally across different healthcare systems [25], the target should be towards sustainable delivery of acute services for gallbladder patients [26,27]. This has not always been achievable with the redistribution of resources during the COVID-19 pandemic, additional pressures upon emergency surgery resources, and complexities arising from the separation of elective and emergency hospital sites requiring restructuring of specific services. Although an ambulatory pathway for acute cholecystitis patients existed within our trust prior to the pandemic, it was necessary to pause this programme to deal with the significant backlog in the elective waiting list. During the period of this study, the only available facility for urgent cholecystectomy was within an emergency surgery setting (in part due to the challenges associated with testing and isolation protocols for patients having surgery at a COVID-secure elective site [28]). As the waiting list backlog continues to reduce and isolation/testing protocols are lifted, it is anticipated that we can change focus to the delivery of ambulatory emergency cholecystectomy within seven days for patients with calculous cholecystitis and reducing the waiting time further for patients with gallstone pancreatitis.

One of the main strengths of our approach to the cholecystectomy waiting list has been the utilisation of a mobile theatre environment (a large transportable trailer with all the necessary inbuilt facilities of a regular operating theatre. They come in single or multiple units of different sizes and can be fitted as an extension to any existing hospital structure). This has provided a self-contained operating theatre (including anaesthetic room and a patient recovery area) within our hospital site but remote to the main theatre complex. This facility became active during the second quarter of the present analysis and contributed around 26% of the overall workload delivered in this project (Table 2). This facility was fully protected from the influence of other factors within the hospital, and, aside from the surgical and anaesthetic team, it was staffed by team members provided externally from the NHS Hospital Trust. Although this facility was utilised on a pre-existing hospital site, it acted in an independent manner (aside from the admission facilities that were needed) and could be modified to work in other environments as an independent ‘surgical hub’ [22]. However, it is important to consider that the approach to the utilisation of ‘surgical hubs’ will not be a ‘one-size-fits-all’ model, and this form of approach represents only one of the potential mechanisms to facilitate the required increases in capacity.

The present results were largely achieved by completing four to five cholecystectomies on each all-day operating list. This is below the target of six cholecystectomies per all-day operating list outlined in the ‘Get It Right First Time” (GIRFT) template for elective recovery [16]. The reasons for this were multi-factorial but largely due to the more complex nature of some cases secondary to the long waiting times due to the COVID-19 pandemic. Although specific operative difficulty grading was not collected in this series [29], long-wait cases with a previous history of cholecystitis operated on during the early phases of the study were anecdotally more challenging than the cases operated on during the later stages. This is believed to be due to recurrent episodes of mild cholecystitis (not always necessitating hospital admission) for these cases during the waiting period. Despite this, it has been possible to provide objective evidence of the effectiveness of this approach upon surgical waiting times whilst maintaining appropriate quality standards. 

The ongoing support of surgical training has been a key discussion surrounding the recovery of services following COVID-19 [30]. In the current series, surgical trainees acted as the primary surgeon in 27.1% of cases and would have performed a significant proportion of many more cases. This has demonstrated that surgical training can be continued within a surgical hub model while maintaining satisfactory outcomes as detailed above.

Limitations of the current study include a lack of data collection regarding difficulty grading for cholecystectomies performed during the period analysed. This would have potentially enabled analysis of any differences in case mix (in terms of surgical difficulty) between the surgical sites. Although the methodology deployed in this study has proven to be extremely effective within our service, these results may not be directly applicable to all hospital trusts nationally or internationally. However, this study does provide objective evidence of what is potentially achievable to significantly reduce waiting lists using cholecystectomy as a worked example. 

## 5. Conclusions

This study has demonstrated the utility of a multi-modality approach to reducing patient waiting time specifically related to laparoscopic cholecystectomy. This form of approach which has utilised multiple hospital sites, mobile operating theatre environments, and seven-day working could be deployed in various settings involving this form of ‘high-volume, low-complexity’ cases to reduce waiting lists. In order to meet the demands placed upon healthcare providers to reduce the backlog following COVID-19, this form of innovative approach will be vital to the delivery of effective services.

## Figures and Tables

**Table 1:** Waiting list categories.

Category	Expected Waiting Time
1a	Under 24 h
1b	Under 72 h
2	Under 1 month
3a	Under 3 months (high priority)
3b	Under 3 months (moderate priority)
4a	After 3 months (high priority)
4b	After 3 months (moderate priority)
4c	After 3 months (low priority)

**Table 2:** Study characteristics for patients who underwent a procedure during the study period.

Characteristic	All (*N* = 657)
Age, median (IQR) years	49 (39–59)
Female sex, n (%)	494 (75.2)
Initial USS diagnosis, n (%)	
* Biliary colic*	380 (57.8)
* Cholecystitis*	228 (34.7)
* Pancreatitis*	30 (4.6)
* * *Gallbladder polyp*	19 (2.9)
ASA Grade, n (%)	
* 1*	192 (29.2)
* 2*	410 (62.4)
* 3+*	55 (8.4)
Waiting list category, n (%)	
* 2*	70 (10.7)
* 3a*	244 (37.1)
* 3b*	122 (18.6)
* 4a–c*	221 (33.6)
Waiting time, median (IQR) days	194 (91–359)
Elective admission, n (%)	628 (95.6)
Site, n (%)	
* Site 1 + 2*	53 (8.1)
* Site 3*	269 (40.9)
* Site 4*	77 (11.7)
* Mobile Theatre*	176 (26.8)
* Private sector*	82 (12.5)
Surgeon, n (%)	
* Consultant*	479 (72.9)
* Registrar*	133 (20.2)
* SHO*	45 (6.9)
Surgery duration, median (IQR) minutes	63 (51–81)
Excision, n (%)	
* Total*	630 (95.9)
* Subtotal*	20 (3.3)
* Abandoned*	7 (1.1)
LOS, median (IQR) days	0 (0–1)
Clavien-Dindo grade, n (%)	
* 2*	19 (0.03)
* 3a*	5 (0.01)
* 3b*	6 (0.01)
* 4a*	1 (>0.01)
30-day readmissions, n (%)	20 (3.0)
30-day mortality, n (%)	0 (0.0)

N/n: number, %: percentage, IQR: interquartile range, USS: ultrasound scan, ASA: Association of American Anaesthesiologists, SHO: Senior House Officer, LOS: length of hospital stay.

## Data Availability

The data presented in this study are available on request from the corresponding author. The data are not publicly available due to ongoing audits and accelerated service adaptation and improvement.
